# *Helitron*-like transposons contributed to the mating system transition from out-crossing to self-fertilizing in polyploid *Brassica napus* L.

**DOI:** 10.1038/srep33785

**Published:** 2016-09-21

**Authors:** Changbin Gao, Guilong Zhou, Chaozhi Ma, Wen Zhai, Tong Zhang, Zhiquan Liu, Yong Yang, Ming Wu, Yao Yue, Zhiqiang Duan, Yaya Li, Bing Li, Jijun Li, Jinxiong Shen, Jinxing Tu, Tingdong Fu

**Affiliations:** 1 National Key Laboratory of Crop Genetic Improvement, National Center of Rapeseed Improvement in Wuhan, Huazhong Agricultural University, Wuhan, 430070, China

## Abstract

The mating system transition in polyploid *Brassica napus* (AACC) from out-crossing to selfing is a typical trait to differentiate it from their diploid progenitors. Elucidating the mechanism of mating system transition has profound consequences for understanding the speciation and evolution in *B. napus*. Functional complementation experiment has shown that the insertion of 3.6 kb into the promoter of self-incompatibility male determining gene, *BnSP11-1* leads to its loss of function in *B. napus*. The inserted fragment was found to be a non-autonomous *Helitron* transposon. Further analysis showed that the inserted 3.6 kb non-autonomous *Helitron* transposon was widely distributed in *B. napus* accessions which contain the *S* haplotype *BnS-1*. Through promoter deletion analysis, an enhancer and a putative cis-regulatory element (TTCTA) that were required for spatio-temporal specific expression of *BnSP11-1* were identified, and both might be disrupted by the insertion of *Helitron* transposon. We suggested that the insertion of *Helitron* transposons in the promoter of *BnSP11-1* gene had altered the mating system and might facilitated the speciation of *B. napus*. Our findings have profound consequences for understanding the self-compatibility in *B. napus* as well as for the trait variations during evolutionary process of plant polyploidization.

Polyploidization drives speciation and diversification in flowering plants. Its impact on plant genomes has primarily been thought of as ‘genomic shock’ accompanied by rapid and extensive genomic and epigenomic changes^1^,^2^,^3^,^4^. In this process, duplicated genes (whole genome duplication, WGD) can be lost, retained or maintained as duplicates, often undergoing subfunctionalization and neofunctionalization^5^,^6^. As a result, polyploids often show different phenotypic traits than their presumed progenitors with respect to morphology, ecology, cytology, and physiology; these new phenotypes may contribute to speciation, adaption to the environment and enhancement of the utility of polyploids for agriculture^7^,^8^,^9^,^10^. The molecular mechanisms underlying the evolution of these novel phenotypes remain largely unknown.

In *Brassica*, mating system transition is particularly well suited to studying the molecular mechanism of phenotypic trait variations in polyploids. The three basic diploid species *B. rapa* (AA, 2n = 20), *B. oleracea* (CC, 2n = 18) and *B. nigra* (BB, 2n = 16) are self-incompatible, but all three cultivated allotetraploids, *B. napus* (AACC, 2n = 38), *B. carinata* (BBCC, 2n = 34) and *B. juncea* (AABB, 2n = 36) are self-compatible, indicating that the mating system has evolved from outcrossing to self-fertilization (selfing) in association with polyploid formation. In many flowering plants, loss of self-incompatibility (SI) caused the transition of mating systems from outcrossing to self-fertilization, with a significant impact on the evolution of these species^11^. Selfing can increase homozygosity and cause inbreeding depression in offspring, however it also confers advantages such as reproductive assurance when pollinators or mates are scarce and higher efficiency of gamete transmission than out-crossing^12^,^13^,^14^,^15^. The molecular mechanism of mating system transition has also been a major focus in evolutionary biology.

Self-incompatibility in the Brassicaceae is controlled sporophytically by the multi-allelic *S* locus (i.e., pollen SI phenotype is determined by the diploid genotype of the pollen-producing parent)^16^. An *S* locus consists mainly of two genes *SP11*/*SCR (S*-locus protein 11/*S*-locus cysteine rich protein) and *SRK (S*-locus receptor kinase) determining recognition specificity in pollen^17^,^18^ and stigma^19^,^20^, respectively. The *S* locus is also called the ‘*S* haplotype’ as the *S*-locus genes are transmitted to progeny as one unit^21^. *S* haplotypes of *Brassica* can be divided into two classes. Class-II haplotypes are generally recessive to class-I haplotypes in pollen, but they are co-dominant in the stigma^19^. When pollen and stigma carry the same *S* haplotype of *Brassica*, self-incompatible interaction occurs, leading to the arrest of ‘self’ pollen at the stigma surface^22^. Any mutations in genes involved in female specificity, male specificity or downstream signaling pathways could cause the loss of SI. Recent studies in wild Brassicaceae species demonstrated that the evolution of self-compatibility (SC) tends to be driven by mutations in the male rather than the female components^11^, such as in *Arabidopsis thaliana*, *Leavenworthia alabamica, Capsella rubella*, *Arabidopsis kamchatica* and others^21^,^23^,^24^,^25^,^26^. However, in domesticated *Brassica* species, female self-compatible mutations are more frequent^11^, such as in *B. napus* (mutations of *SRK*^27^), *B. rapa* (mutations of *SRK*^28^ and *MLPK*, M-locusprotein kinase^29^) *and B. oleracea* (mutation of *SRK*^30^). As the determinant of SI recognition specificity in pollen, *SP11*/*SCR* gene expression was tightly regulated and coordinated with stamen development to confer a successful self-incompatible reaction. In *B. rapa*, class II *BrSP11-60* was found to be expressed mainly in the anther tapetum, while transcripts of class I *SP11*/*SCR* genes were detected clearly both early in anther tapetum development and late in pollen development by RNA gel blot analysis^18^,^22^,^31^,^32^,^33^. The class I SP11/SCR protein product was present in the tapetum and pollen, but late in anther development the SP11/SCR protein was mainly localized in the pollen coat^34^. Also, the dominance relationship between SI alleles (*SP11*/*SCR* gene) was proposed to be responsible for SC in the polyploid species^26^,^27^,^35^,^36^.

Cultivated *B. napus* is a self-compatible species, although it carries *S* haplotypes. In *B. napus* cultivar ‘Westar’, a dominant *S* haplotype *BnS-1* derived from *B. rapa* haplotype *BrS-47* on the A genome and a recessive *S* haplotype *BnS-6* derived from the *B. oleracea* haplotype *BoS-15* on the C genome were identified, with both *SP11*/*SCR* genes having lost their function^27^. An insertion of a DNA element of 3606 bp in the promoter region of *BnSP11-1* gene on the A genome was responsible for the SC of ‘Westar’^27^,^37^. But how the insertion was generated and affected the evolution of *B. napus* remains unknown. In this study, by analyzing the 3.6 kb fragment inserted in the promoter of *BnSP11-1*, a non-autonomous *Helitron* transposon was identified. Further analysis showed that this *Helitron* transposon did not appear in *B. rapa (BrS-47*) but was widely distributed in *B. napus (BnS-1*), which indicated that it moved into the promoter of *BnSP11-1* gene after formation of the polyploid species *B. napus*. By promoter deletion analysis, we found the insertion had disrupted the enhancer sequence and other *cis*-regulatory elements required for the spatio-temporal specific expression of *BnSP11-1*. We propose that the movement of the *Helitron* type transposon caused the transition of mating system, with a significant impact on the origin and evolution of *B. napus*. Our results yield insight into the complex mechanisms of both loss of self-incompatibility in *B. napus* and phenotypic trait variation in polyploid plants.

## Results

### Validation of the role of the inserted fragment in *BnSP11-1* gene

**Figure 1:**
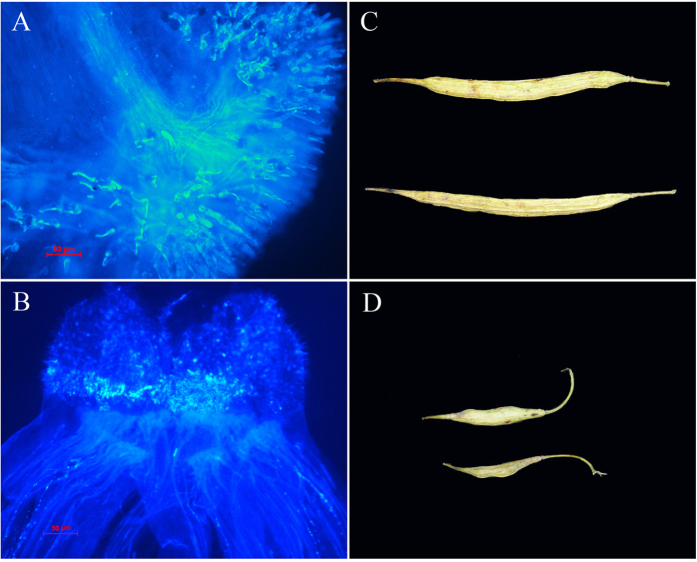
Pollination assays of the transgenic line ‘W-3’ and the wild type ‘Westar’. (**A**) ‘Westar’ pollen placed on the stigma of ‘W-3’ shows compatible interaction, with many pollen tubes penetrating the stigma; (**B**)‘W-3’ pollen placed on the stigma of ‘Westar’ shows self-incompatible reaction, with no pollen tubes observed; (**C**) Pods set many seeds in the compatible pollination; (**D**) Pods set few seeds in the incompatible pollination. Bars = 50 μm in (**A,B**).

To confirm the role of the inserted 3606 bp element in *BnSP11-1* for the SC of ‘Westar’ proposed by Okamoto *et al.*^27^ and Tochigi *et al.*^37^, the *SP11/SCR* gene in *BrS-47 (BnS-1* was derived from *BrS-47*) was used to complement the function of *BnSP11-1* in ‘Westar’ (Additional Information: Figure S1A). RT-PCR analysis showed that *BrSP11-47* transcripts can be detected in mature buds of ten transgenic plants and all of them were self-incompatible to varying degrees (Additional Information: Figure S1C). T1 progeny plants were also self-incompatible and the trait co-segregated with the introduced DNA. Transgenic line ‘W-3’ showed a higher level of *BrSP11-47* transcripts and stronger self-incompatibility (setting only several seeds by self-pollination) than other lines, so it was used for further analysis (Additional Information: Figure S1B,C). Pollination assays showed that when the pollen of ‘Westar’ was applied to the stigma of ‘W-3’, compatible interaction occurs with many pollen tubes penetrating the stigma and resulting in normal pod set. When the pollen of ‘W-3’ was applied to the stigma of ‘Westar’, self-incompatible reaction occurs, no pollen tubes were observed, and the pods set few seeds (Fig. 1). These results showed that *BnSRK-1* had normal function and the non-functional *BnSP11-1* gene (with the 3606 bp insertion) on the A genome was responsible for the SC of ‘Westar’ in *B. napus*.

### *Helitron* like transposon identification in the promoter of the *BnSP11-1* gene and evolutionary analysis

**Figure 2:**
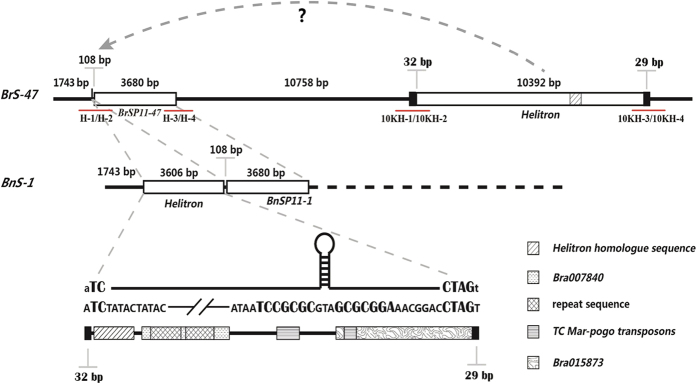
Two non-autonomous *Helitron* type transposable elements inserted independently in the *S* haplotypes *BrS-47* and *BnS-1*. The *Helitron* in *BrS-47* lies downstream of the *SP11/SCR* gene but is absent from *BnS-1*. The *Helitron* in *BnS-1* lies in the promoter region of the *SP11/SCR* gene. Both *Helitrons* shared similar boundaries, small hairpin structures, and embedded (captured) sequences.

The 3606 bp DNA element inserted in the promoter of the *BnSP11-1* gene (Genbank accession AB270773) was analyzed to explore how it was generated and affected the evolution of *B. napus*. The inserted fragment showed no sequence similarity to known transposable elements. However, manually it was found to contain all the structural characteristics of a novel family of putative rolling circle transposable elements in eukaryotes, termed *Helitron*^38^. Like other *Helitrons* reported, the 3606 bp element was inserted precisely between the nucleotides 5′-A and T-3′, and did not cause duplication of the insertion site sequence. Furthermore, this fragment starts with 5′-TC, ends with 3′-CTAG, and is accompanied by a predicted small hairpin structure near the 3′ end (Fig. 2). As it lacks sequences similar to DNA helicase and RPA-like proteins which are necessary for being autonomous, we concluded that the insertion in *BnSP11-1* is a non-autonomous *Helitron* type transposable element.

Plant *Helitrons* often capture gene fragments during their movement^38^. By BLASTN analysis of the inserted element in BRAD (*Brassica* database, http://brassicadb.org/brad/), we found that positions 542 to 1333 bp showed almost 90% similarity to *Bra007840* and positions 2292 to 3606 bp were similar to *Bra015873*. Interestingly, positions 114 to 474 bp showed 85% similarity to a BAC sequence (Genbank accession AB180899.1) that contains the *SP11*/*SCR* gene of *S* haplotype *BrS-47*. As *S* haplotype *BnS-1* was derived from *BrS-47*, the BAC sequence was further analyzed. To our surprise, another *Helitron* type transposable element with a length of 10393 bp was identified downstream of the *BrSP11/SCR-47* gene (Fig. 2). These two *Helitron* type transposable elements showed sequence similarity at the termini (32 bp at the 5′ terminal and 29 bp at the 3′ terminal), shared the same predicted small hairpin structure near the 3′ end, and even captured a similar small gene fragment (positions 114 to 474 bp of the 3606 bp fragment) during their movement (Fig. 2).

To detect the distribution of these two *Helitron* transposable elements in *B. napus*, 123 inbred lines were collected and analyzed. Three primer combinations: H-1/H-2 and H-3/H-4 flanking the 5′ and 3′ end of the *Helitron* transposable element (Fig. 2), and SpeS1-5/SpeS1-6 which can specifically amplify the *BnSP11-1* intron were designed (Additional Information: Table S2). All three pairs of primers showed amplification in 85 lines but not in the remaining 38 lines (Additional Information: Table S3), indicating that the *Helitron* transposable element inserted in *BnSP11-1* was widely distributed in *B. napus* having the *S* haplotype *BnS-1*, and moved into the promoter after formation of *B. napus*. In addition, two primer combinations: 10KH-1/10KH-2 and 10KH -3/10KH-4 flanking the 5′ and 3′ end of the 10393 bp *Helitron* (Fig. 2; Additional Information: Tables S1 and S2, Figure S3) did not show amplification in any of the 123 *B. napus* inbreds (Additional Information: Table S3), indicating that it moved away from downstream of *BrSP11/SCR-47* gene after the formation of *B. napus*.

### Disruption of *cis*-regulatory elements in the promoter of *BnSP11-1*

**Figure 3:**
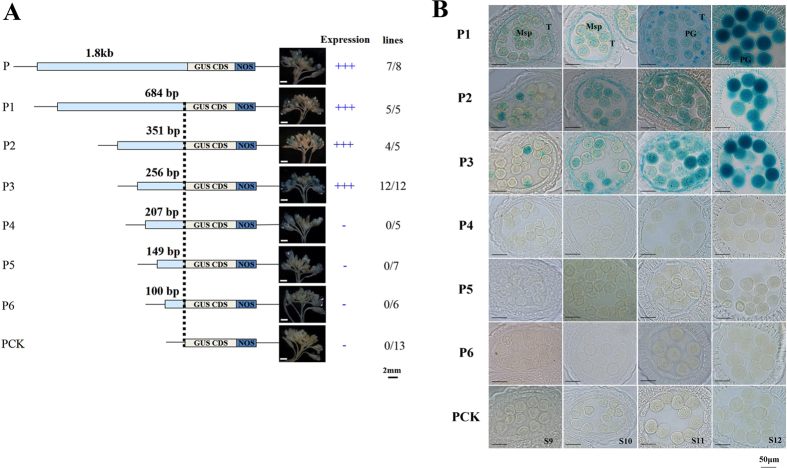
*BnSP11-1* promoter deletion analysis. (**A**) Promoter deletion constructs (P1-GUS to P6-GUS), the control construct PCK and summary of GUS staining results. Numbers indicate positions relative to the *BnSP11-1* translation start site. Photographs were taken after 16 h GUS incubation. Relative expression of each promoter construct in stamen is represented by +(positive) or −(negative). ‘Lines’ column indicates the number of individual transformants displaying stamen GUS activity over total number of transformants analyzed; Bars = 2 mm. (**B**) Representative GUS staining results for semi-thin section from stage 9 to stage 12 of lines transgenic for the indicated *BnSP11-1* promoter deletion constructs and the control construct PCK. T, tapetum; Ms, microsporocyte; Tds, tetrads; Msp, microspore; PG, pollen grain; Bars = 50 μm.

Overlapping PCR technology was used to isolate the promoter of the *BnSP11-1* gene that did not contain the *Helitron*. A fragment of 1851 bp 5′-upstream of the translation initiation site was obtained and used to drive *GUS* (β-glucuronidase) gene expression in *Arabidopsis* (P-GUS). GUS staining results showed that the promoter was functional (Additional Information: Figure S2). To identify and characterize *cis*-regulatory elements involved in promoter strength and specificity, promoter deletion analysis was conducted. Six 5′-deletion promoter fragments linked to *GUS* were introduced into *Arabidopsis* (P1-GUS to P6-GUS, Fig. 3A). GUS staining can be detected in older buds in P1-GUS to P3-GUS but not in P4-GUS to P6-GUS under a stereo microscope (Fig. 3A). By semi-thin section analysis, no obvious difference in GUS staining (in the tapetum, microspores and mature pollen from stage 9 to stage 12) was observed in P1-GUS (−681 bp), P2-GUS (−351 bp) and P3-GUS (−256 bp) transgenic plants (Fig. 3B). However, GUS staining cannot be detected in P4-GUS (−207 bp), P5-GUS (−149 bp), P6-GUS (−100 bp) fusions and the control construct PCK at any stage of anther development (Fig. 3B). It indicated that the region from −256 bp (P3-GUS) to −207 bp (P4-GUS) contained *cis*-regulatory elements responsible for the spatial and temporal expression patterns of the *BnSP11-1* gene.

**Figure 4:**
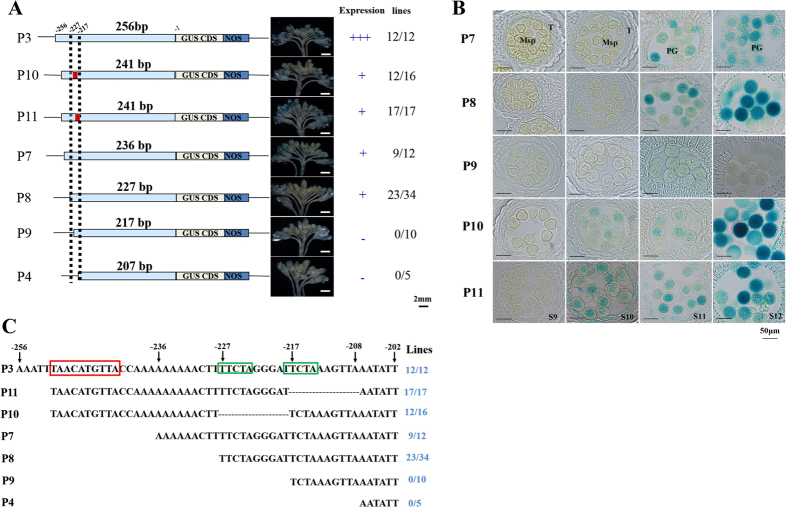
Further deletion analysis of the *BnSP11-1* promoter. (**A**) Promoter deletion constructs (P7-GUS, P8-GUS, P9-GUS, P10-GUS and P11-GUS) and representative GUS staining results. Numbers indicate positions relative to the *BnSP11-1* translation start site. Photographs were taken after 16 h GUS incubation. Relative expression of each construct in stamen is represented by +(positive) or −(negative). ‘Lines’ indicate numbers of individual transformants displaying stamen GUS activity over total number of transformants analyzed; Bars = 2 mm. (**B**) Representative GUS staining results for semithin sections from stages 9 to 12 in transgenic lines of P7, P8, P9, P10 and P11. T, tapetum; Msp, microspore; PG, pollen grain; Bars = 50 μm. (**C**) Summary of *BnSP11-1* promoter deletion analysis results. By sequence alignment of the deleted promoter fragments, a palindromic sequence (TAACTAGTTA, red box) and a putative *cis*-element (TTCTA, green box) responsible for the spatial and temporal expression patterns of *BnSP11-1* were identified.

Further, P7-GUS to P9-GUS were constructed based on the region from −256 bp (P3-GUS) to −207 bp (P4-GUS) (Fig. 4A). GUS staining was detected in P7-GUS and P8-GUS, but not in P9-GUS (Fig. 4A), showing that the region near −227 bp to −217 bp has *cis*-regulatory elements responsible for the spatial and temporal expression of *BnSP11-1*. Compared with P3-GUS, P7-GUS and P8-GUS initiated gene expression at later stages: GUS staining was detected weakly at stage 9 and 10, but strongly at stage 11 and 12 (Fig. 4A,B). Therefore, the 20 bp sequence from −256 to −237 bp was proposed to determine early stage expression strength of *BnSP11-1*. A putative 10 bp palindromic sequence (TAACTAGTTA) was identified, which was considered an enhancer (Fig. 4C).

To identify the *cis*-regulatory elements exactly, P10-GUS and P11-GUS were constructed (Fig. 4A). Unexpectedly, P10-GUS and P11-GUS had almost the same staining patterns as P7-GUS and P8-GUS (Fig. 4A,B). We speculated that both the deleted regions in P10-GUS (−227 bp to −218 bp) and P11-GUS (−217 bp to −208 bp) were necessary for early stage *BnSP11-1* expression. Also, they played redundant roles in pollen grain expression at late stages. By analyzing the sequences in P10-GUS, P11-GUS and P9-GUS, a putative *cis*-element (TTCTA) located in both deleted regions was identified (Fig. 4C). We also concluded that the *Helitron* type transposable element inserted 108 bp upstream of the translation initiation site has disrupted enhancer sequences and other *cis*-regulatory elements responsible for the normal expression of *BnSP11-1*.

## Discussion

Unlike their progenitors *B. rapa* (AA) and *B. oleracea* (CC) that are self-incompatible and mainly used as vegetables, cultivated allotetraploid *B. napus* (AACC) genotypes are self-compatible and mainly grown for seed harvest. The mating system transition from SI to SC has therefore been a key event which may have contributed to *B. napus* speciation, increasing its adaptation to the environment and its utility for agriculture. In this study, we found the mating system transition from SI to SC in *B. napus* was caused by a *Helitron*-like transposon that inserted in the promoter of *BnSP11-1* gene. The inserted *Helitron*-like transposon was widely distributed in *B. napus* containing the *S* haplotype *BnS-1*. By promoter deletion analysis we found the insertion of the *Helitron*-like transposon had disrupted the enhancer sequence and other *cis*-regulatory elements responsible for the normal expression of *BnSP11-1* gene.

TEs (transposable elements) are one of the major components of plant genomes and also one of the major drivers of plant genome evolution^39^,^40^. Interspecific hybridization, accompanied by ‘genomic shock’, has been proposed to induce bursts of transposition attributable to the interaction of merged genomes, as first proposed by McClintock^1^. Beyond their considerable contribution to genome structure, they also influence gene expression^2^,^41^,^42^,^43^. *Helitrons*, as eukaryotic DNA transposable elements, are predicted to amplify by a rolling-circle mechanism and constitute about 2% of the *A. thaliana* genome^38^. In maize, most *Helitrons* are non-automonous elements with truncated pseudogenes and/or mobile elements that are considered to be responsible for rich in transpecific genetic diversity and the loss of function of some nuclear genes^44^,^45^,^46^,^47^. In *B. rapa*, an insertion of a *Helitron* was reported to be responsible for the yellow seed trait of cultivar *yellow sarson*^48^. Both the DNA elements inserted independently in the promoter region of the *BnSP11-1* gene and downstream of the *BrSP11-47* gene were non-autonomous *Helitron* transposons, which contain no transposase gene(s) but some truncated pseudogenes. The *BnSP11-1* and *BrSP11-47* associated *Helitrons* showed sequence similarity at the termini (32 bp at the 5′ terminal and 29 bp at the 3′ terminal), the same predicted small hairpin structure near the 3′ end and even a similar captured gene fragment (position 114 to 474 of the 3606 bp fragment) (Fig. 2). As the transposition mechanism of *Helitrons* is unclear, we could not extend our hypothesis to propose that the *BnSP11-1* associated *Helitron* might be resulted from the movement of the *BrSP11-47-*associated one during *B. napus* speciation and evolution. By analyzing 123 inbred lines of *B. napus*, we found that all 85 lines which contain the *S* haplotype *BnS-1* also had the 3606 bp *Helitron* transposon inserted in the promoter of *BnSP11-1*, but none of them have the *Helitron* inserted downstream of *BrSP11-47* (Additional Information: Table S3). As a result, the movement of the *Helitron* transposon caused the mating system transition from SI to SC of the polyploid species *B. napus* (Fig. 1). Our results provided evidence that transposons played a key role for the evolution of polyploid species.

Mutations disabling male specificity (*SP11*/*SCR* gene) of the SI system are expected to be more strongly selected in wild species as mutant pollen grains are more easily transmitted to the offspring than mutant ovules during the pollination process, contrasting with the prevalent mutations disabling female specificity in domesticated species^11^,^49^,^50^,^51^,^52^. However, in the present investigation, *Helitron* transposon insertion in *BnSP11-1* gene (male self-compatible mutation) was responsible for SC in ‘Westar’. *SP11/SCR* expression was tightly regulated and was under the control of a gene regulatory network involved in anther development. Shiba *et al.*^34^ suggested that the sporophytic and gametophytic expression patterns of the *SP11/SCR* gene are controlled by different *cis*-regulatory elements. The intact *BnSP11-1* gene promoter can drive *GUS* gene expression in anther tapetum and microspores at stage 9, reaching maximum expression at stage 12, when anthers contain tricellular pollen grains and appear bilocular after degeneration and breakage of the septum below the stomium^53^ (Additional Information: Figure S2). In *B. rapa*, the highly conserved 192-bp upstream region was sufficient to drive the unique expression of *BnSP11-9*^34^. However, we observed delayed *GUS* expression in tapetum and pollen in P7-GUS (−236 bp) and P8-GUS (−227 bp) with detailed semi-thin section analysis (Fig. 4B). Further, we identified a putative 10 bp palindromic sequence (TAACTAGTTA) considered an enhancer, and a putative *cis*-element (TTCTA), that played redundant roles in pollen grain expression at late stages (Fig. 4C). Revealing the molecular mechanism of *SP11/SCR* gene expression and regulation might provide a foundation for mating system transition research in the Brassicaceae species.

There are two *S* haplotypes that determined the SI reaction located on A and C genome, respectively in *B. napus*, both of them might contribute to the mating system transition. Here we report that *Helitron*-like transposon insertion on A genome was responsible for mating system transition in ‘Westar’ (Figs 1 and 2). While on C genome, expression of the recessive *BnSP11-6* gene cannot be detected, though the DNA sequence was intact, which might be suppressed by the dominant nonfunctional *BnSP11-1* gene on A genome^27^. In the Brassicaceae, complex dominance interactions among *S*-haplotypes have been reported^54^,^55^,^56^, and the underlying molecular mechanism have been partially revealed in *B. rapa*^31^,^32^,^57^. If the reported mechanism of dominance relationship between SI alleles in *B. rapa* contributed to the mating system transition in allotetraploid *B. napus* needs to be explored. In allotetraploid *A. kamchatica*, the degradation of male components was responsible for the loss of SI, both homeologous copies of the *SP11/SCR* gene ought to have lost their function by interspecific crosses analysis and also the dominance interactions of the SI alleles may be involved^26^. Zhai *et al.*^36^ proposed that besides the mutation of *SP11/SCR* genes and dominance relationship between SI alleles, other factor(s) independent of the *S* locus are involved in the SC of some accessions in *B. napus*. Although, the precise mechanism of dominance relationship between SI alleles is still unclear, and it was proposed to be responsible for the SC in the polyploid species^26^,^27^,^35^,^36^.

**Figure 5:**
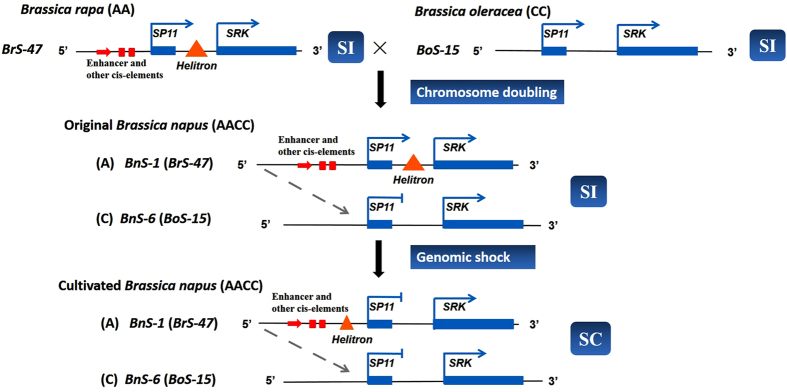
A proposed model for the origin and formation of *B. napus*. In the original *B. napus* plants, *BrS-47* was dominant over *BoS-15* in pollen, which may suppress the expression of recessive *SP11/SCR* gene located on the C genome. As the *SP11/SCR* and *SRK* genes located on the A genome can be expressed normally, the original *B. napus* plants were inferred to be self-incompatible. The genome of the newly formed amphidiploid plants was unstable, with transposable elements playing a pivotal role in providing variation for genome reorganization. A *Helitron* transposon disrupted *cis*-regulatory elements responsible for the normal expression of *BnSP11-1* and conferred.

Based on those results, a speculative model for the origin and speciation of *B. napus* was proposed (Fig. 5): After the formation of the original *B. napus* plants by inter-specific hybridization between *B. rapa* and *B. oleracea*, the expression of the recessive *SP11/SCR* gene located on the *B. oleracea*-derived C genome was suppressed by an unknown mechanism. However, the *SP11/SCR* and *SRK* genes on the *B. rapa*-derived A genome were expressed normally, therefore the original *B. napus* plants were inferred to be self-incompatible. The movement of a *Helitron* transposon disrupted the enhancer sequence and other *cis-*regulatory elements responsible for the normal expression of *BnSP11-1* gene, conferring SC and permitting newly formed *B. napus* plants to produce seeds. SC shows particular advantages when out-crossing mates are scarce^50^,^51^, such as they may be for a newly formed polyploidy. So, we suggested that the movement of *Helitron* transposons and the dominance interactions between the *S* alleles might coordinately contribute to the origin and speciation of *B. napus* by changing the mating system from cross pollination to self-pollination. Our findings provide insight into the self-compatibility of *B. napus* as well as other trait variations associated with the evolutionary process of plant polyploidization.

## Methods

### Plant Materials and Growth Conditions

The wild type self-compatible *B. napus* line ‘Westar’, its transgenic plants and *B. rapa* line ‘9-117’ with *S* haplotype *BrS-47* were grown in the greenhouse with a 16/8 h day/night photoperiod and day/night temperatures of 22 °C/15 °C. *A. thaliana* plants (ecotype Columbia) were grown at 22 °C, 16/8 h light/dark in the greenhouse.

### Investigation of SI phenotype

Self-incompatibility phenotype was measured as follows^58^: when three to five flowers were set on the major inflorescence, the major inflorescence and two or three secondary ramifications were bagged for self-pollination after removing the apical buds artificially. Every two days, bags were slipped gently in order to assure enough self-pollination. Self-seeds were produced by bud-pollination. About two weeks later, bags were removed to allow the seeds to develop. After seedpods were mature, the number of seeds produced was counted, and self-compatibility index (SCI) was calculated as the ratio of number of seeds to number of flowers^59^. Plants with SCI ≥ 2 were referred as self-compatible and plants with SCI < 2 were considered as self-incompatible^58^.

### Promoter Region of *BnSP11-1* and Promoter Deletion Constructs

Overlapping PCR technology was used to isolate the clean promoter fragment (no *Helitron* type transposable element contained) of the *BnSP11-1* gene from ‘Westar’. Primer combination BnS1PRO-3/BnS1PRO-4 (5′-ACGCGTCGACAGCTTCACTCTTGGACTGTC-3′/5′-TAACAATCATTATAAATACATATCCAACAGAAGTTGCGTA-3′) was used to amplify the 5′- flanking region of the *Helitron* type transposable element and primer combination BnS1PRO-5/BnS1PRO-2 (5′-TACGCAACTTCTGTTGGATATGTATTTATAATGATTGTTA-3′/5′-TCCCCCGGGGATTCAGAAAAGTGATAAAAGATTC-3′) was used to amplify the 3′-flanking region of the *Helitron* type transposable element. *SalI* and *SmaI* restriction sites were added to the 5′ and 3′ ends of the primers BnS1PRO-3 and BnS1PRO-2 respectively. A fragment of 1851 bp of the 5′-upstream region of translation initiation site was obtained. A cassette containing the GUS coding region followed by the nopaline synthase polyadenylation signal from pBI101 (CLONTECH, Palo Alto, CA) was subcloned into the binary vector pCAMBIA 2300^60^ with restriction enzymes *Hind III* and *EcoRI* to construct promoter-GUS fusions. The amplified fragments were subcloned into the modified binary vector pCAMBIA 2300^60^ to yield the 1851-bp *SP11-1* promoter-GUS construct. Based on the resultant promoter sequence of *BnSP11-1*, a series of deletion constructs (P1-GUS to P11-GUS) were generated. BnS1PRO-2 (5′-TCCCCCGGGGATTCAGAAAAGTGATAAAAGATTC-3′) which contains a *SmaI* restriction site to the 3′ end was used as the antisense primer to amplify the deletion promoter fragment for all deletion constructs. Sense primers for each deletion construct were listed as follows: P1-GUS: (5′-ACGCGTCGACGACACACCATCACCACTTCTTT-3′), P2-GUS: (5′-ACGCGTCGACCTTTTAGACCTCCTTAATAGCCTG-3′), P3-GUS: (5′-ACGCGTCGACAAATTTAACATGTTACCAAAAAAA-3′), P4-GUS: (5′-ACGCGTCGACAATATTTGGACCCGTTAATCTC-3′), P5-GUS: (5′-ACGCGTCGACTTTAGTTAAAAAATCTGTTTTACG-3′), P6-GUS: (5′-ACGCGTCGACTAATGATTGTTAACAAGGAAAC-3′), P7-GUS: (5′-ACGCGTCGACAAAAAAACTTTTCTAGGGATTCT-3′), P8-GUS: (5′-ACGCGTCGACTTCTAGGGATTCTAAAGTTAAATA-3′), P9-GUS: (5′-ACGCGTCGACTCTAAAGTTAAATATTTGGACC-3′), P10-GUS: (5′-ACGCGTCGACTAACATGTTACCAAAAAAAAACTTTCTAAAGTTAAATATTTGGACCCG-3′) and P11-GUS: (5′-ACGCGTCGACTAACATGTTACCAAAAAAAAACTTTTCTAGGGATAATATTTGGACCCGTTAATCTCGTTG-3′). All the sense primers contain *SalI* restriction site at the 5′ end. All the amplified deletion promoter fragments were subcloned independently into the modified binary vector pCAMBIA 2300^60^ to yield the P1-GUS to P11-GUS constructs. The modified binary vector pCAMBIA 2300^60^ was used as the negative control.

### Vector Construction of *BrSP11-47* gene and Plant Transformation

Overlapping PCR technology was used to clone the promoter sequence and the coding sequence (CDS) of *BrSP11-47* (GenBank accession no. AB180899) from a *B. rapa* line ‘9-117’ with homozygous *BrS-47*. Primer combination S1E1/S1E2 (5′-TCCCCCGGGTACGACCTGCTGATATTCTCC-3′/5′-ATCAGATTAGCTTCCACTTCTTGAATATGACCTGAAACG-3′) was used to amplify the promoter region and the first exon and primer combination S1E3/S1E4 (5′-CGTTTCAGGTCATATTCAAGAAGTGGAAGCTAATCTGAT-3′/5′-GGGTTACCCTAACACAATTTACATACACAAGAATAA-3′) was used to amplify the second exon of *BrSP11-47. SmaI* and *BstEII* restriction sites were added to the 5′ and 3′ ends of the primers S1E-1 and S1E-4 respectively. Finally, a fragment of 2345 bp containing the promoter region and the CDS of *BrSP11-47* was obtained. This fragment was then subcloned into the binary vector pCAMBIA2301^60^ to yield the 2301-1 + 4 construct.

The construct 2301-1 + 4 was introduced into *A. tumefaciens* GV3101 host cells. Plant transformation was carried out following the method of Dun *et al.*^61^. The transformed plants with roots were subsequently transplanted in experimental plots from which T1 seeds were harvested. DNA from transgenic plants was analyzed by PCR, combining the primers S1E1 (5′-TCCCCCGGGTACGACCTGCTGATATTCTCC-3′) and PC2301R (5′-GCAACAGGATTCAATCTTAAGAA-3′) designed from the sequence of the nopaline synthase polyadenylation signal present in the vector to verify the presence of the *SP11* transgene.

### Pollination Assay

Floral buds of the *B. napus* plants were emasculated one day before anthesis to avoid pollen contamination. Pollination was performed the next day. Some pollinated pistils were left to set seeds. The rest were cut at the peduncle 16 hours after pollination, fixed for 2 h in ethanol: acetic acid (3:1), softened in 1 N NaOH at 60 °C for 1.5 h, and stained with 0.01% (w/v) decolorized aniline blue for 2.5 h in 2% (w/v) K3PO4. Pistils were gently squashed onto a microscopic slide glass by placing the cover glass over the pistils. Samples were examined under a fluorescence microscope (Ax 10, Zeiss).

### GUS assay

All promoter-GUS constructs were introduced into *Arabidopsis* wild-type plants (ecotype Columbia) by *Agrobacterium*-mediated transformation. GUS activity was visualized by staining different stage flowers in the T3 generation of homozygous transgenic lines, overnight in X-Gluc solution^62^, and then tissues were cleared in 75% (v/v) ethanol. Treated flower buds were observed and photographed under a stereomicroscope, then embedded in Technovit 7100 resin (HeraeusKulzer, http://www.heraeus.com/) as described previously by Zhu *et al.*^63^. Afterwards, transverse sections of the anthers approximately 12 μm thick were cut from the embedded blocks using a Leica Ultracut R ultra-microtome (Leica). The sections were photographed under a microscope and the anther development stages were determined^53^.

### RT-PCR and qRT-PCR

Total RNA was extracted using a plant mini RNeasy kit (Qiagen). Five micrograms of RNA was DNase-treated using a DNA-free kit (Ambion, http://www.ambion.com). First-strand cDNA synthesis was performed using a SuperScript kit (Gibco BRL, http://www.invitrogen.com). The reverse transcription products were used as templates for PCR to examine the expression of *BrSP11-47* in transgenic plants. Real-time RT-PCR was also performed using a Bio-Rad IQ5 with SYBR Green detection (http://www.bio-rad.com/). Primer combination RT-SCR1-L/RT-SCR1-R (5′-TGTTTCATATTCATCGTTTCAGG-3′/5′-CTCTTGTCCATACCCTTCGAATA-3′) was used for RT-PCR analysis. Primer combination RT-SCR1-1/RT-SCR1-2 (5′-GCTAATCTGATGAATCCGTGCG-3′/5′-TTTGTGCATTCGCAACGTGG-3′) was used for Real-time RT-PCR analysis. Actin (Gene-Bank accession no.: AF111812) was amplified with RAC1-P3/RAC1-P4 and used as an internal control to normalize transcript levels for all the expression analyses.

## Additional Information

**How to cite this article**: Gao, C. *et al.* *Helitron*-like transposons contributed to the mating system transition from out-crossing to self-fertilizing in polyploid *Brassica napus* L. *Sci. Rep.* **6**, 33785; doi: 10.1038/srep33785 (2016).

## Supplementary Material

Supplementary Information
