# Supplementary material for: Helitron-like transposons contributed to the mating system transition from out-crossing to self-fertilizing in polyploid Brassica napus L
Source: Sci Rep. 2016 Sep 21;6:33785. doi: 10.1038/srep33785 (PMC5030654; doi:10.1038/srep33785)
Supplement: Supplementary Information [file srep33785-s1.pdf]

Helitron-like transposons contributed to the mating system transition from out-crossing to self-fertilizing in polyploid *Brassica napus* L.

Changbin Gao[+], Guilong Zhou[+], Chaozhi Ma[*], Wen Zhai, Tong Zhang, Zhiquan Liu, Yong Yang, Ming Wu, Yao Yue, ZhiqiangDuan,Ya Li, Bin Li, Jijun Li, Jinxiong Shen, Jinxing Tu&Tingdong Fu

Additional Information

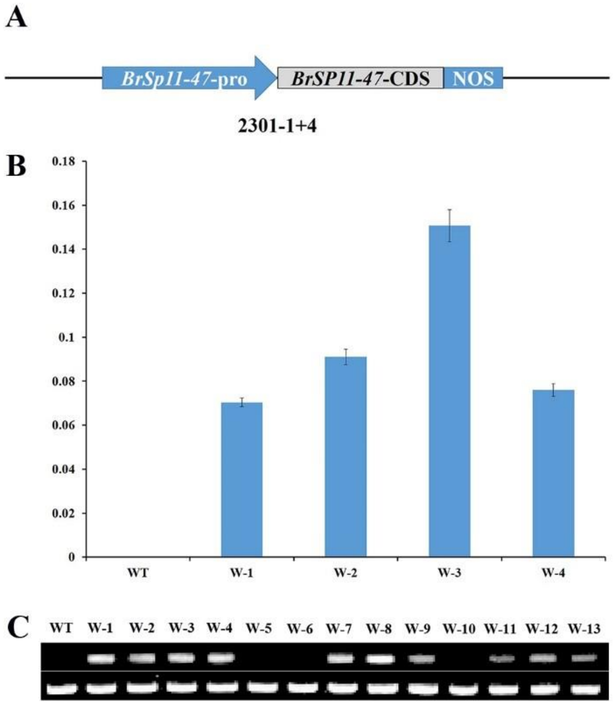

Figure S1. The expression vector 2301-1+4 and detection of *BrSP11-47* transcripts in transgenic plants by real-time RT-PCR and RT-PCR analysis. A. Schematic illustration of construct 2301-1+4 was used to transform ‘Westar’. B. Real-time RT-PCR analysis of *BrSP11-47* expression in four transgenic plants (W-1 to W-4). Stamens were used for RNA extraction. C. RT-PCR analysis of *BrSP11-47* gene expression in transgenic planwasts (W-1 to W-13). Mature buds were used for RNA extraction.

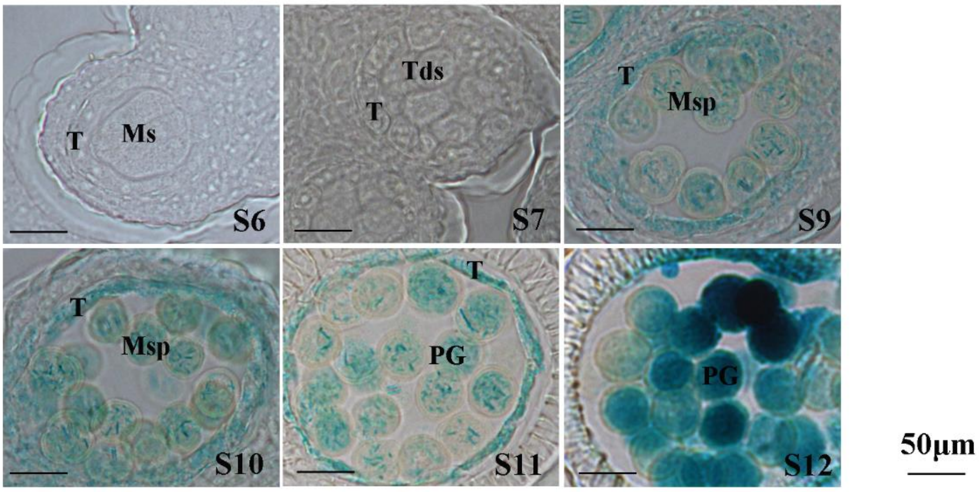

Figure S2. GUS assay of *BnSP11-1*. After removal of the *Helitron* with overlapping PCR in the *BnSP11-1* promoter, it can drive GUS gene expression from stages 9 to 12 in the tapetum, microspores and pollen grains. Bars = 50 μm;

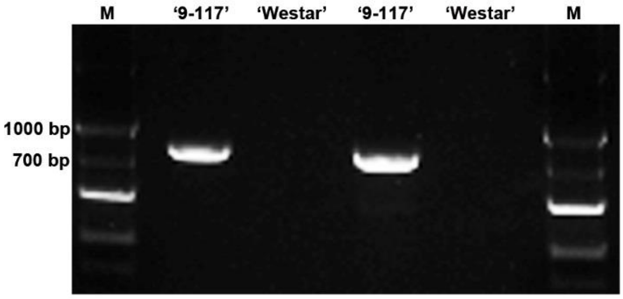

Figure S3. Amplification results of the primer combinations 10KH-1/10KH-2 and 10KH-3/10KH-4 in '9-117' and 'Westar'. M, Marker. Sequencing result showed that primer combination 10KH-1/10KH-2 can produce 810 bp fragment, primer combination 10KH-3/10KH-4 can produce 773 bp fragment, both fragments showed 100% sequence similarity with the published BAC sequence (Genbank accession AB180899.1).

Materials	Phenotypes	SCI
WT	SC	12.31
W-1	SI	0.028
W-2	SI	0.73
W-3	SI	0.009
W-4	SI	0.02
W-5	SC	9.22
W-6	SC	13.07
W-7	SI	0.093
W-8	SI	0.015
W-9	SI	0.098
W-10	SC	8.35
W-11	SI	0.16
W-12	SI	0.054
W-13	SI	1.183

Table S1. Investigation of SI phenotypes of the 13 transgenic plants. SC, self-compatibility; SI, self-incompatibility; SCI, self-compatibility index; WT as a control.

Primers	Nucleotide sequence	Annealing temp (℃)	Length of fragment
SpeS1-5	TCTGAATCATGAAATCTGCTGT	54	412 bp
SpeS1-6	TTAAAAGGATTCTGCAAAGTTCT		
Helitron-1	TACTCTCCTTCCCGTCTCCTG	58	941 bp
Helitron-2	CTTCTTGCAACCCAAACACTG		
Helitron-3	ATAAAATGTTGGATCTGTCGAAGC	55	764 bp
Helitron-4	CCCTGCAGAAAATTCTTGTTTATC		
10KHelitron-1	TGCGTTAAGATTTGTTCATGTGC	55	810 bp
10KHelitron-2	ATCACAAACACGAATCGAGACAA		
10KHelitron-3	GGCACGAATGTAAAATCAGGTTAA	55	773 bp
10KHelitron-4	TCGAGAGTCAAGAGATGTAGGTCG		

Table S2. The primers used for detection of *S* haplotype *BnS-1* and *Helitron* transposable elements.

40

S. No.	Materials	SpeS1-5/SpeS1-6	H-1/H-2	H-3/H-4	10KH-1/10KH-2	10KH-3/10KH-4
1	9-455-1	1	1	1	0	0
2	9-1222-1	1	1	1	0	0
3	9-1859-1	0	0	0	0	0
4	9-1908-1	0	0	0	0	0
5	9-1908-2	0	0	0	0	0
6	9-2925-1	1	1	1	0	0
7	9-2925-2	1	1	1	0	0
8	9-3100-1	1	1	1	0	0
9	9-3101-1	1	1	1	0	0
10	9-3102-1	1	1	1	0	0
11	9-3103-1	1	1	1	0	0
12	9-3109-1	0	0	0	0	0
13	9-3111-1	1	1	1	0	0
14	9-3112-1	0	0	0	0	0
15	9-3115-1	0	0	0	0	0
16	9-3116-1	1	1	1	0	0
17	9-3122-1	1	1	1	0	0
18	9-3124-1	1	1	1	0	0
19	9-3133-1	1	1	1	0	0
20	9-3139-1	1	1	1	0	0
21	9-3144-1	1	1	1	0	0
22	9-3144-2	1	1	1	0	0
23	9-3145-1	1	1	1	0	0
24	9-3146-1	0	0	0	0	0
25	9-3147-1	1	1	1	0	0
26	9-3148-1	1	1	1	0	0
27	9-3149-1	1	1	1	0	0
28	9-3150-1	1	1	1	0	0
29	9-3151-1	1	1	1	0	0
30	9-3152-1	1	1	1	0	0
31	9-3153-1	1	1	1	0	0
32	9-3154-1	1	1	1	0	0
33	9-3156-1	1	1	1	0	0
34	9-3157-1	0	0	0	0	0
35	9-3313-1	0	0	0	0	0
36	9-3329-1	0	0	0	0	0
37	9-3340-1	1	1	1	0	0
38	9-3368-1	1	1	1	0	0
39	9-3401-1	0	0	0	0	0
40	9-3409-1	1	1	1	0	0

41	9-3427-1	0	0	0	0	0
42	9-3432-1	1	1	1	0	0
43	9-3456-1	1	1	1	0	0
44	9-3770-1	1	1	1	0	0
45	9-3857-1	0	0	0	0	0
46	9-3892-1	0	0	0	0	0
47	9-3899-1	1	1	1	0	0
48	9-3900-1	0	0	0	0	0
49	9-3902-1	1	1	1	0	0
50	9-3916-1	1	1	1	0	0
51	9-3922-1	0	0	0	0	0
52	9-3933-1	0	0	0	0	0
53	9-4015-1	1	1	1	0	0
54	9-4027-1	1	1	1	0	0
55	9-4057-1	1	1	1	0	0
56	9-4077-1	0	0	0	0	0
57	9-4084-1	0	0	0	0	0
58	9-4087-1	0	0	0	0	0
59	9-4089-1	1	1	1	0	0
60	9-4111-1	1	1	1	0	0
61	9-4120-1	1	1	1	0	0
62	9-4130-1	1	1	1	0	0
63	9-4310-2	0	0	0	0	0
64	9-4328-1	0	0	0	0	0
65	9-4338-1	0	0	0	0	0
66	9-4343-1	1	1	1	0	0
67	9-4344-1	1	1	1	0	0
68	9-4383-1	1	1	1	0	0
69	9-4389-1	1	1	1	0	0
70	9-4400-1	0	0	0	0	0
71	9-4443-1	0	0	0	0	0
72	9-4459-1	0	0	0	0	0
73	9-4496-1	1	1	1	0	0
74	9-4503-1	1	1	1	0	0
75	9-4507-2	1	1	1	0	0
76	9-4529-1	0	0	0	0	0
77	9-4533-1	0	0	0	0	0
78	9-4536-1	1	1	1	0	0
79	9-4540-1	1	1	1	0	0
80	9-4545-1	1	1	1	0	0
81	9-4549-1	1	1	1	0	0
82	9-4555-1	1	1	1	0	0
83	9-4572-1	1	1	1	0	0
84	9-4576-1	1	1	1	0	0

85	9-4578-1	1	1	1	0	0
86	9-4581-1	1	1	1	0	0
87	9-4583-1	1	1	1	0	0
88	9-4600-1	1	1	1	0	0
89	9-4619-1	1	1	1	0	0
90	9-5009-1	0	0	0	0	0
91	9-5019-1	1	1	1	0	0
92	9-5029-1	0	0	0	0	0
93	9-5036-1	1	1	1	0	0
94	9-5044-1	1	1	1	0	0
95	9-5049-1	1	1	1	0	0
96	9-5209-1	0	0	0	0	0
97	9-5210-1	0	0	0	0	0
98	9-5211-1	1	1	1	0	0
99	9-5221-1	1	1	1	0	0
100	9-5227-1	1	1	1	0	0
101	9-6015-1	1	1	1	0	0
102	9-4603-1	1	1	1	0	0
103	9-4604-1	1	1	1	0	0
104	9-4610-1	0	0	0	0	0
105	9-4613-1	1	1	1	0	0
106	9-4614-1	1	1	1	0	0
107	9-4614-13	1	1	1	0	0
108	9-4617-1	0	0	0	0	0
109	9-4626-1	1	1	1	0	0
110	9-4631-1	1	1	1	0	0
111	9-6018-1	0	0	0	0	0
112	9-6019-7	0	0	0	0	0
113	9-hu12-1	1	1	1	0	0
114	Co1	1	1	1	0	0
115	Co2	1	1	1	0	0
116	Co3	1	1	1	0	0
117	Co4	1	1	1	0	0
118	Co5-1	1	1	1	0	0
119	Co7	1	1	1	0	0
120	Co8-1	1	1	1	0	0
121	Co9-1	0	0	0	0	0
122	Co10-1	0	0	0	0	0
123	9-4635-1	1	1	1	0	0

1 represented amplification; 0 represented No amplification.

Table S3. Amplification data with *BnS-1* and *Helitron* transposable elements specific

primers.
